# Barriers and facilitators affecting the implementation of substance use screening in primary care clinics: a qualitative study of patients, providers, and staff

**DOI:** 10.1186/s13722-018-0110-8

**Published:** 2018-04-09

**Authors:** Jennifer McNeely, Pritika C. Kumar, Traci Rieckmann, Erica Sedlander, Sarah Farkas, Christine Chollak, Joseph L. Kannry, Aida Vega, Eva A. Waite, Lauren A. Peccoralo, Richard N. Rosenthal, Dennis McCarty, John Rotrosen

**Affiliations:** 1 Department of Population Health, New York University School of Medicine, 550 First Avenue, VZ30 6th Floor, New York, NY 10016 USA; 2 Division of General Internal Medicine, Department of Medicine, New York University School of Medicine, 550 First Avenue, New York, NY 10016 USA; 3 Greenfield Health and Department of Psychiatry, Oregon Health and Science University, 9450 SW Barnes Suite 100, Portland, OR 97225 USA; 4 Department of Psychiatry, New York University School of Medicine, One Park Avenue, 8th Floor, New York, NY 10016 USA; 5 Division of General Internal Medicine, Department of Medicine, Icahn School of Medicine at Mt. Sinai, One Gustave L. Levy Place, New York, NY 10029 USA; 6 Department of Psychiatry, Icahn School of Medicine at Mt. Sinai, 1090 Amsterdam Avenue, New York, NY 10025 USA; 7 OHSU-PSU School of Public Health, Oregon Health and Science University, 3181 SW Sam Jackson Park Road, Portland, OR 97239 USA

**Keywords:** Substance use disorders, Drug abuse screening, Alcohol screening, Alcohol abuse, Drug abuse, Primary care, Qualitative research

## Abstract

**Background:**

Alcohol and drug use are leading causes of morbidity and mortality that frequently go unidentified in medical settings. As part of a multi-phase study to implement electronic health record-integrated substance use screening in primary care clinics, we interviewed key clinical stakeholders to identify current substance use screening practices, barriers to screening, and recommendations for its implementation.

**Methods:**

Focus groups and individual interviews were conducted with 67 stakeholders, including patients, primary care providers (faculty and resident physicians), nurses, and medical assistants, in two urban academic health systems. Themes were identified using an inductive approach, revised through an iterative process, and mapped to the Knowledge to Action (KTA) framework, which guides the implementation of new clinical practices (Graham et al. in J Contin Educ Health Prof 26(1):13–24, 2006).

**Results:**

Factors affecting implementation based on KTA elements were identified from participant narratives. *Identifying the problem:* Participants consistently agreed that having knowledge of a patient’s substance use is important because of its impacts on health and medical care, that substance use is not properly identified in medical settings currently, and that universal screening is the best approach. *Assessing barriers:* Patients expressed concerns about consequences of disclosing substance use, confidentiality, and the individual’s own reluctance to acknowledge a substance use problem. Barriers identified by providers included individual-level factors such as lack of clinical knowledge and training, as well as systems-level factors including time pressure, resources, lack of space, and difficulty accessing addiction treatment. *Adapting to the local context:* Most patients and providers stated that the primary care provider should play a key role in substance use screening and interventions. Opinions diverged regarding the optimal approach to delivering screening, although most preferred a patient self-administered approach. Many providers reported that taking effective action once unhealthy substance use is identified is crucial.

**Conclusions:**

Participants expressed support for substance use screening as a valuable part of medical care, and identified individual-level as well as systems-level barriers to its implementation. These findings suggest that screening programs should clearly communicate the goals of screening to patients and proactively counteract stigma, address staff concerns regarding time and workflow, and provide education as well as treatment resources to primary care providers.

**Electronic supplementary material:**

The online version of this article (10.1186/s13722-018-0110-8) contains supplementary material, which is available to authorized users.

## Background

Alcohol and drug use are among the top ten causes of preventable death in the United States [1–4], but substance use disorders (SUDs) are greatly under-treated in the specialty addiction treatment system [5], and under-recognized in medical settings [6, 7]. Screening for alcohol use in adult primary care settings is recommended by the United States Preventive Services Task Force (USPSTF) and ranks as the third highest prevention priority for adults in the U.S [8–13]. The U.S. Surgeon General’s report on addiction recommends screening for other drug use as well as alcohol, which is the current approach of federally-funded ‘screening, brief intervention, and referral to treatment (SBIRT)’ programs [14, 15]. Yet despite over a decade of concerted efforts to integrate substance use screening and interventions into mainstream medical care [16], primary care patients are rarely screened, assessed or treated for SUDs [6, 7, 17–22].

There is a rich literature on barriers to implementing screening for substance use in primary care [17–20]. In today’s busy primary care practices, the time required for screening, challenges of integrating it into the clinical workflow, and poor quality of screening are the primary barriers. Most of the research documenting barriers to screening has been conducted with primary care physicians, but current primary care practice emphasizes a team-based approach and patient-centered care, and successful screening approaches typically involve other members of the care team performing the screening [23, 24]. The perspectives of non-physician clinical staff and patients are thus critical to developing effective implementation strategies, but have been underrepresented in the screening literature.

We sought to gain an understanding of substance use screening from a diversity of clinical stakeholders, as part of a multi-site study of the National Institute on Drug Abuse (NIDA) Clinical Trials Network (CTN). The overarching goal of this CTN study is to implement substance use screening using the NIDA CTN Common Data Elements (CDEs) in primary care clinics. The CDEs are validated substance use screening tools that have been selected for integration into electronic health records (EHRs) based on their accuracy and feasibility in medical settings [25, 26].

The conceptual model for the study is the Knowledge to Action (KTA) framework, which informs the selection and implementation of new clinical practices. Implementation models and frameworks can both guide the introduction of new practices, and inform our understanding of why the introduction of a given practice or intervention may succeed or fail [27]. While there is now a wealth of experience with implementing SBIRT through demonstration projects, it remains challenging to explain why certain approaches may have been successful in some settings but not in others, or to arrive at a set of key elements that are generally needed for the effective introduction of screening into health care settings [24]. The KTA framework was developed by implementation researchers based on a synthesis of 31 theories for planned action [28, 29]. It is considered a process model, meaning that it can be used to describe or guide the translation of research into practice [27]. Our study activities focus on the KTA ‘action cycle,’ and consist of adapting, implementing, and evaluating the use of substance use screening tools. This paper presents results of the initial phase of the larger CTN study, and used qualitative methods to identify current substance use screening practices, barriers to screening, and recommendations for its implementation in primary care clinics.

## Methods

### Design

Focus groups and individual interviews with clinical stakeholders were conducted to inform the development of EHR-integrated substance use screening tools, and strategies for their implementation in primary care clinics participating in the CTN study. A qualitative approach was used because we sought to gain an understanding of the attitudes of medical providers, clinical staff, and patients toward screening, and to elicit recommendations for introducing screening into these clinical sites. Focus groups elicited group norms and attitudes from each category of stakeholders, while individual interviews gathered more detailed information about the logistics of introducing EHR-integrated screening, and gave an in-depth understanding of primary care provider attitudes and perceived barriers. The study was approved by the Institutional Review Boards of NYU School of Medicine, Icahn School of Medicine at Mt. Sinai, and Oregon Health and & Science University.

### Setting

The majority of data collection was done in one New York City health system that serves as the lead site for this CTN study, and has two participating primary care clinics. To gain additional insight from practitioners with greater SBIRT experience, we also conducted interviews at a second study site located in Portland, Oregon. Oregon introduced screening and brief intervention for alcohol and drug use in 2013 as an incentive measure for Coordinated Care Organizations (CCOs), under the state’s health care transformation [30, 31]. Additionally, some of the Oregon participants for this study had interacted with a federally-funded SBIRT training program based at Oregon Health & Science University.

These urban, academic medical centers were chosen as sites for the CTN study because they have large networks of primary care practices, serve as training sites for multidisciplinary healthcare providers, are geographically diverse, and use Epic EHR software (Epic Systems Corporation). Epic is one of the most widely-utilized EHRs in the U.S. [32], and we chose Epic-using health systems in order to facilitate knowledge transfer between the study sites and enable future broad dissemination of the clinical tools that we plan to develop. Participating clinics within each health system were selected with a goal of providing a diversity of primary care practice settings. The two New York City sites were internal medicine clinics; one teaching practice that serves primarily a safety net patient population (NY-1), and one faculty practice (NY-2). The Portland (OR) sites were a family medicine and an internal medicine practice. At the time of the study, none of the sites had initiated systematic screening for alcohol or drug use.

### Participants

Focus groups and individual interviews were conducted between June 2015 and February 2016, with a total of 67 participants. *Medical providers* were primary care providers (PCPs); medical assistants (MAs); or registered nurses (RNs). Our medical provider sample included both faculty and residents; although non-MD primary care providers (including nurse practitioners and Doctors of Osteopathy) were eligible to participate, our sample was composed entirely of MDs. *Patients* were individuals currently receiving care in one of the participating adult primary care clinics. A total of 11 focus groups were conducted at the New York clinics with faculty PCPs (two groups), residents (one group), MAs (two groups), RNs (one group), and patients (five groups). Because medical residents provided care at only one of the clinics, only one session was held with residents. At the smaller of the two New York clinics, RNs and MAs were combined into a single group. At the Oregon site, scheduling difficulties made it unfeasible to schedule and recruit participants for focus groups, and so only individual interviews were conducted.

Eight individual interviews were conducted with PCPs in Oregon (n = 6) and New York (n = 2). Individuals were selected for interviews using a critical case sampling approach [33, 34]. In New York, the interviews occurred after the focus groups, and were used to provide more in-depth exploration of questions or themes that were raised during the focus group sessions. The two PCPs selected for individual interviews were full-time faculty providers who had participated in a focus group and articulated good understanding of the clinic’s operations. In Oregon, PCPs were individuals who were selected because of their SBIRT or health informatics experience, and were current full-time clinical faculty (n = 4) or primary care clinician informaticists (n = 2) at the study clinics.

To encourage participation, focus groups were held during regular scheduled meeting times and interviews were conducted on-site and during regular clinic hours. For focus groups with medical staff, all eligible individuals were invited by email to attend. For focus groups with patients, clinical staff distributed fliers with information about the group to patients presenting for care, and directed individuals who were interested to speak with a research assistant. All adult patients who were fluent in English were eligible to participate. Patients’ medical providers were not involved in recruitment, and were not informed regarding participation of their patients in the study. All participants received an IRB-approved written information sheet, and the interviewer verbally summarized its content and solicited questions prior to each session. All participants and gave verbal consent to the interview and audio recording. Participants were not asked to review transcripts or provide feedback on our findings.

### Methodology

The semi-structured interviews sought to evaluate constructs from the Knowledge to Action (KTA) framework that guide selection and implementation of new clinical practices; in this case, the inclusion of substance use screening in primary care clinics. Interview guides were developed by the CTN study Lead Investigator and followed the themes from the action phase of the KTA framework: identifying the problem; assessing barriers; and adapting to the local context. Interview guides (included as Additional file 1) covered views on the purpose, value, and acceptability of screening for substance use, current screening practices, opinions regarding providers’ knowledge about substance use, and recommendations about the frequency and content of screening as well as how to integrate it into the clinical workflow. Interview guides for medical providers, staff, and patients covered the same general topics, but were tailored to the participant group (for example, interviews with patients addressed answering screening questions, while interviews with providers addressed delivering screening, receiving results, and clinical workflow). For medical providers, focus group and individual interview guides were similar, but individual interviews gave emphasis to personal experiences and attitudes, while focus groups emphasized current practices in the clinic and group attitudes regarding screening.

### Data collection

Focus groups and interviews were conducted between June and November, 2015. The New York focus groups and interviews were conducted on-site at the clinics by the Lead Investigator (JM), who is a practicing PCP at a different New York City medical center. The interviews in Oregon were conducted by a psychologist (TR) who was the local site lead. Both interviewers have experience conducting qualitative health services research. Interviews were conducted in English. The average length of the focus groups and interviews was 45–60 min and all sessions were audio recorded. For each focus group, at least one member of the research staff observed the group and took field notes, while for individual interviews field notes were written by the interviewer following the session. Participants were given a small monetary amount for participation (staff received $50, patients received $20). Payments were calibrated to the amounts that are typically used for research in these clinical sites, and were higher for staff because they may have needed to take time away from paid clinical work in order to participate. Following each interview or focus group, participants were asked to complete a form collecting demographic information.

### Data analysis

Interviews were transcribed verbatim from the audio recording, leaving out any names. A research assistant who attended all of the focus groups verified the transcripts by comparing them to the audio recordings. Transcripts were entered into Atlas ti (7.0) software for data management and analysis. Field notes were reviewed when needed by the interviewers (JM and TR) to facilitate recall, but were not included in the analysis.

Two researchers (PC and ES) reviewed each focus group and interview transcript. The initial review was done independently, and then the codes that each researcher had developed were discussed and redundancies were eliminated to arrive at an initial codebook. Transcripts were analyzed using thematic analysis; an inductive approach designed to identify and examine emerging themes from conceptual data that involves coding qualitative data using a list of themes found in the transcripts [35]. Initial codes and categories were developed to identify theoretically important concepts of the KTA. Emerging themes were mapped to the domains and constructs of the KTA after multiple readings of the transcripts. Several discussions were conducted among researchers (PCK, ES and JM) involved in the coding and analysis about the appropriateness of each code. Disagreements around codes, themes and subthemes were resolved by discussion among the team members and going back to the original transcripts and field notes. The final coding scheme consisted of 6 primary themes and 46 subthemes for provider transcripts and 4 primary themes and 38 subthemes for patient transcripts. Three researchers (PCK, ES and JM) concluded that thematic saturation was reached when no additional themes related to the KTA domains were emerging from the data.

Using the jointly developed codebook, ES coded all transcripts. Blinded to the coding of ES, PK coded a randomly selected 13 transcripts (20%) to establish inter-rater reliability. The Kappa coefficient for inter-rater reliability was 0.75 which is interpreted as excellent in qualitative literature [36]. In a final step, the themes and subthemes were aggregated into matrices, organized by the KTA domains, containing the most representative quotes from each interview. Matrix analyses visually display the range of related responses for each theme, to ensure that both majority and outlier responses are considered [37]. For example, the matrix for the theme ‘identifying the problem’ had columns named for each subtheme, and rows named for each interview type; cells were filled with quotes from the interviews that captured the subtheme.

## Results

Demographic characteristics of the 67 participants are included in Table 1. Among the PCP participants, 29 were faculty and 5 were internal medicine residents. Two PCPs participated in both a focus group and an individual interview. A total of 15 medical assistants and 3 registered nurses participated. Most of the patient participants were over 45 years of age and female, and were primarily recruited from the New York teaching practice (NY-1 clinic).

**Table 1:** Characteristics of the 67 participants

Characteristic	Total N = 67	MDs N = 34	MAs and RNs N = 18	Patients N = 15
Age—mean (SD)	46 (SD = 12)	39 (11)	40 (SD = 9)	52 (SD = 13)
Age range	27–72	27–68	27–56	28–72
Age group				
26–35	21	14	6	1
36–45	19	10	6	3
46 +	25	8	5	11
Missing	2	2	1	0
Sex				
Female	49	21	14	12
Male	18	13	4	3
Hispanic				
No	50	30	10	10
Yes	14	2	8	4
Missing	3	2	0	1
Race				
Caucasian	23	19	2	1
Asian	14	13	1	0
Black	15	0	6	9
Other	10	0	6	4
Missing	5	2	3	1
Medical specialty	N/A		N/A	N/A
Internal medicine		29		
Family medicine		2		
Missing		3		
Patients seen per week	N/A		N/A	N/A
0–50		18		
51–100		11		
> 100		1		
Missing		4		

Our results are described in three broad categories, based on the KTA framework. The first category, ‘identifying the problem,’ contains themes related to the value of screening and current practices. The second, ‘assessing barriers,’ includes themes related to individual-level and systems-level barriers to screening. The third category, ‘adapting to the local context,’ consists of recommendations from participants about implementing screening.

### Identifying the problem

All stakeholder groups felt that identifying substance use is important for providing appropriate medical care. At the time of the study, none of the participating clinics had implemented a systematic approach to alcohol or drug screening, though they did have systems in place for tobacco screening. In this setting, participants felt that unhealthy alcohol and drug use among patients may go unidentified.

#### Left to the discretion of the provider, screening is not regularly done

Providers said that they asked about substance use during some visits, particularly during a patient’s initial visit to the clinic or when pursuing a specific health complaint that may be alcohol- or drug-related. Drawbacks to this approach noted by providers were that the information may not be accurate, because patterns of substance use can change over time, or that patients may not feel comfortable disclosing substance use in an initial meeting with the provider. Participants noted that some patients may never be asked about alcohol or drug use, because screening depends on the provider, the patient’s other medical problems, and the amount of time available.

#### Screening has value for medical care

Overwhelmingly, patients and providers agreed that substance use screening has value in primary care. Providers stated that knowing about a patient’s substance use is critical to understanding their overall state of health, and that it impacts their care for other conditions.

Some PCPs talked about substance use being similar to other social determinants of health that impact disease risk and response to treatment, ‘like whether they have a job and a place to live’ (NY-1 Faculty MD focus group). Many providers discussed specific ways in which knowledge of substance use could affect their diagnoses, prescribing decisions, and care plans. As summarized by one PCP:

I do think it is probably both important and necessary, because I guess for two reasons. One, we are a point of intervention for something that might not otherwise be identified or discussed in somebody’s life. But I also think it can be destructive. And I think people often don’t know where to talk about it. So I think it’s important to ask to give people that opportunity, but also to better understand who you’re working with and whether that’s part of why it’s difficult to care for other identified disease processes.

NY-1 Faculty MD interview

Similar views were held by MAs and patients, who agreed that substance use affects overall health, and that medical providers need to know about substance use in order to provide good care. As stated by one MA: “Well, that would give the doctor information on how to take care of the patient, direct them to where they have to go if they need help, and help them with their care” (NY-2 MA focus group). Patients discussed that it was important for their medical providers knowing about substance use, to allow them to make accurate diagnoses and provide appropriate treatment. As one patient said, “And, yes, I want them to know everything about me so I can get the best possible diagnosis I can possibly get” (NY-1 Patient focus group).

In addition to providing overall knowledge about the patient’s health, providers and patients also talked about specific ways in which screening for substance use adds value to the clinical encounter. Screening was felt to be a way of signaling to patients that talking about substance use is permissible, thus opening the door to a conversation that would not have otherwise have happened. Participants also noted that identifying substance use is a necessary first step toward helping patients with substance problems. Providers stated that primary care visits present an opportunity to identify and intervene upon unhealthy use. One expression of these views is from a PCP focus group:

Some patients actually want to be helped. And they may not ask for help unless you bring it up. So sometimes that’s why they’re actually here. But they won’t tell you that. [Another participant, agreeing] “Patients don’t…I mean, most patients won’t bring it up themselves.”

NY-1 Faculty MD focus group

Another way in which screening was thought to add value was by providing a ‘teachable moment’ for educating patients about substance use and related harms. This potential benefit was raised by both patients and PCPs, who felt that patients may not understand that their substance use is a health risk until they are specifically asked about it. As stated by a patient:

Some people don’t know how to ask for help. Or sometimes they don’t even realize that they need help. So maybe just talking about it with their doctor, you might not even realize how much you really do drink or how much you really do smoke unless someone asks you, oh, so how many drinks, you know… Like, how many drinks would you say that you had in a week? And if you’re somebody that’s like, oh, I don’t have a problem. But if you think about it and be like, well, I have about ten drinks a week, you may begin to think like maybe I do have a problem. So, you know, it keeps that… It makes doctors and patients actually talk about their issues….

NY-1 Patient focus group

### Assessing barriers to screening for substance use

Participants identified both individual-level and systems-level barriers to implementing substance use screening in primary care settings. Individual-level factors for patients included feeling uncomfortable disclosing substance use because they fear a negative reaction from their provider, concerns about confidentiality, and not being ready to discuss their substance use. Individual-level factors for providers included their knowledge and capacity to respond effectively to substance use. Systems-level factors included limitations imposed by the clinic’s physical environment and lack of time.

#### Individual level barriers

##### Patients worry about how medical providers will react

Patients and providers stated that patients may be uncomfortable disclosing substance use out of fear of being judged by their provider. They noted that the quality of the patient-provider relationship is an important determinant of whether patients will feel comfortable disclosing substance use. This viewpoint is captured in the following patient’s statement:

You know, people are so afraid, I think sometimes of being judged. That sometimes it just creates this impediment in regards to being as forthcoming as you possibly can. I think the relationship [with the physician] is really the foundation…

NY-2 Patient focus group

Some patients expressed concern about unforeseen consequences of screening, if providers were to react negatively to a patient’s disclosure of substance use. One patient speculated that if they felt uncomfortable with their provider’s reaction it could impact their engagement in care, although they did not see this as a barrier for all patients.

Even, like, your doctor, it’s like, well, maybe they’ll look at me differently now. And then I’m uncomfortable coming. And then, so it could create this kind of like spiral of paranoia, you know. And then being uncomfortable and then feeling like, oh, now I have to leave. And like it could not end so well. You know what I mean? But then there’s people who I think would be totally honest.

NY-2 Patient focus group

##### Patients are concerned about having substance use information in the medical record

Patients also expressed concerns about substance use information appearing in their medical record, and how that could affect the care they receive from other providers. Some patients felt that having substance use information in their medical record could potentially impact their job, insurance payments for medical care, and providers’ willingness to prescribe some medications (such as controlled substances). This overall unease with documentation of substance use was captured in the words of one patient:

…with the substance use being judged or, you know, against I guess societal expectations or whatever, I just think that that could also pose…you know, have someone feeling like I’m not too comfortable, you know, with that being in the records.

NY-2 Patient focus group

Providers were sensitive to patients’ concerns about substance use information appearing in the medical record. One of the Oregon physicians with health information technology expertise discussed interacting with other healthcare providers who feel that it may not be appropriate to fully integrate substance use information into the EHR. A number of providers expressed heightened concern about documenting substance use, especially now that patients are often able to view their own medical records. This view is summarized here by one MA, but was similarly expressed by PCPs who worry about how patients will react to seeing substance use documented in their chart.

And it doesn’t say confidential or whatever. So people are very worried. And it actually puts us at major risk when we start documenting all this stuff, that the patients will come back and say.. ‘you said that I drank, you know, five bottles a day and I’m an alcoholic.’ They read these things.

NY-2 MA focus group

##### Substance use is viewed differently from other medical conditions

Patients talked about the stigma of substance use, and some felt that the patient gets blamed for having a substance use disorder. While this was not a theme in the provider interviews, patients were sensitive to how a patient with a substance use disorder could feel accused of bringing the condition upon themselves, and worried about how this would impact their treatment. As expressed by one patient:

I would think it would be something about substance use, because there’s a stigma attached to it. Like, the fact that you maybe had cancer or you had heart disease, like you could say, well, that’s not my fault. Like that’s something that happened to me. And I overcame this, right? As opposed to the way the world looks at substance abuse as ‘this is your fault, you did this to yourself’ type of mentality. So I think that it would definitely be a difference between your previous medical conditions and substance abuse.

NY-2 Patient focus group

##### Patients may not be ready to disclose substance use

Patients and providers discussed that an individual’s own reluctance to acknowledge having a substance use problem, even to themselves, can pose a barrier to screening. Many participants stated that patients need to be ready to be honest with themselves about their substance use before they can be expected to disclose it to their medical providers. A related theme was that patients who are not ready and willing to receive help will not disclose substance use, and for these patients screening may be ineffective. This perspective was summarized by a patient:

And so if a person’s willing to get honest and truthful about where they’re at in their station in life….Then it’s good. But if the person is still addicted and deny their drug use or alcoholism or whatever it is, you know, the screening, I don’t know how effective that would be.

NY-1 Patient focus group

##### Provider knowledge and training

Both faculty and resident PCPs identified knowledge deficits as a barrier to providing screening and interventions to address substance use. Several providers stated that although they received training in addressing other behavioral health conditions, such as smoking cessation and depression treatment, they had less knowledge about alcohol and drug use. As a result, they felt poorly prepared to address substance use when it was identified. Knowledge was discussed as both a lack of education about substance use and as a lack of familiarity with assessing patients and linking them to appropriate treatment. As stated by one faculty PCP:

So that’s a barrier for me, is like my own lack of knowledge of what’s out there or what’s reliable and good, and what the culture of those places is [so] that I can even advise my patients, [for example] ‘Hey, I think this would be a good fit for you because,’ or ‘You might want to try to this first instead because.’ And I think that’s important when somebody is making a choice to try to do something like that.

NY-2 Faculty MD focus group

Although a small number of PCPs in our sample noted that they had received specialized training in substance use and felt comfortable with substance use interventions, they still felt that they would benefit from additional hands-on teaching, such as having a coach to observe their clinical interactions and offer feedback.

Residents in particular expressed discomfort with not knowing how to address a positive screening result, because they do not know how to treat it themselves and are unfamiliar with the treatment resources available in their clinic or in the community. As one resident noted, they may be knowledgeable about treating tobacco use, and feel confident with smoking cessation counseling, but they feel poorly prepared to intervene if a patient has alcohol or drug use.

I think for smoking I have a pretty good idea of like options for my patients. And I counsel them fairly often about it. And, you know, there’s like medication options and like all that sort of stuff. But for alcohol and drugs, like I have no idea like where to send them if they screen positive.

NY-1 Resident MD focus group

Patients themselves also expressed concern that providers may not have the proper training or knowledge to work with patients who report substance use. Some discussed substance use as a condition that requires specialized care, and one patient equated expecting physicians to address substance use with asking a cardiologist ‘about what’s going on with your foot.’ While they were skeptical about doctors being able to intervene, patients did express more confidence in their ability to make referrals for treatment. As one patient stated,

But I don’t believe that they know how to handle that. They would refer you to someone. But I don’t think they would know what to do.

NY-2 Patient focus group

##### PCPs may fail to address a positive screening result

Medical assistants expressed concern that primary care providers would not respond appropriately to patients with substance use problems. This would make them feel less comfortable screening, because they were not confident that it would ultimately help the patient.

Because I feel like if he’ll tell me, a patient, oh yeah, I’m an alcoholic, I need help. Like she said with the depression, here’s a box of tissues, sit outside. And then the doctor doesn’t address it. And he says, ‘I told you I had this problem and nothing was done.’ And I’m sorry, but then we have care coordinators, navigators, like all these resources but they feel nobody’s there to help them. So like we’re in the middle. Then you feel bad. Here they come again. This is the second time you ask me this question, and they didn’t help me the first time so I’m not going to answer. We’ve had that.

NY-2 MA focus group

#### System-level barriers

A number of systems-level barriers were identified by providers. The main barriers identified were lack of space and privacy in the clinic, poor access to treatment for patients with substance use disorders, and the time pressures of primary care visits.

##### Lack of privacy

Medical assistants felt strongly that screening for substance use should be done individually and in a private room. Many reported that they did not have enough privacy in their workspace, and that screening under these conditions would be disrespectful to patients. One MA expressed that screening in their current space would make patients angry.

The space is so tight. And the space is so limited… She used to have…I don’t know if you still do, two chairs in the same room, two patients at the same time, two MAs working. How are you going to ask that kind of question to a patient? They’re going to fly, right, they’re going to be very angry. I mean, that’s something very private.

NY-1 MA focus group

##### Lack of treatment resources for patients with substance use problems

Medical providers stated that better systems are necessary for delivering effective treatment to patients with SUDs, and that this needs to be in place before initiating a screening program. Several providers mentioned that they do not know where to refer patients, and that they lack resources in the clinic to help patients with substance use problems. As one resident expressed, it is discouraging for providers to feel that they cannot connect patients to appropriate care.

And I feel like the limitations on where we can refer people afterwards. Like, well, you don’t quality for inpatient rehab. So I guess, what are we going to do? Try to go to AA? It didn’t really feel like we were providing that much of a service…

NY-1 Resident MD focus group

Most providers and patients discussed substance use treatment as something that happens outside the primary care clinic, and requires referral to a community-based program. However, some providers expressed hope that there would be more primary care-integrated treatment in the future, particularly with the increased use of medication for addiction treatment. This perspective was summarized by one of the Oregon PCPs:

It doesn’t do any good to screen for anything that you don’t have a treatment for or can’t access a treatment for, and I think there is hope as medicated medication assisted treatments expand into primary care. You know, the providers will have more tools available to them. Hopefully, we’ll have more counseling resources as a result of the Affordable Care Act and mental health parity in primary care, but right now most primary care clinics don’t.

OR Faculty MD interview

Providers who worked in clinics that had a social worker or behavioral health specialist saw them as a valuable member of the care team, but stated that insufficient resources are devoted to integrated care for substance use in the primary care setting. Specifically, one provider noted that their clinic has one substance use counselor for ‘several thousand patients in internal medicine,’ and that despite the high level of need, the funding for this position is constantly in jeopardy because the counselor is unable to bill for services.

##### Time

Overwhelmingly, medical providers and staff identified lack of time as a barrier to addressing substance use in primary care. They noted that visits are brief, and that there are usually multiple competing priorities, such as the patient’s chief complaint and other medical conditions. An example was given by one physician:

And I’m thinking of one person in particular who comes in, and by the time we talked about his chronic pain and primary hyper- pulmonary hypertension, neuropathy and fall risk and depression, I rarely have time to get to his substance use screening issues. Even though I’ve been taking care of him for thirteen years now and I know that he has a remote history of alcohol use disorder, I rarely, in a single visit, will get to that.

OR Faculty MD interview

Relatedly, some providers felt that discussing substance use felt like ‘opening a can of worms’ that would be difficult to address. Some suggested that being able to bring patients back for a visit that focused just on substance use would help, but also noted that their schedules were usually so full that this may not be possible. These concerns were expressed by faculty and resident PCPs, and captured here in the words of a resident:

I think one of the other things is we’re a little afraid of substance abuse in the sense of that, if it comes up I feel like in order to really deal with it there are a lot of psychosocial issues that are behind it. Like people have a lot of co-morbid mental illness. And those are just difficult and very time consuming issues for us. So you really need like a dedicated visit when that comes up.

NY-1 Resident MD focus group

### Adapting knowledge to the local context

Participants made a number of recommendations for how to implement screening in their practices. There was broad agreement across stakeholder groups on some aspects of the screening approach, but differences of opinion regarding other aspects, such as who among the clinical staff should deliver screening.

#### What substances should screening address?

##### Screening should encompass tobacco, alcohol and other drugs

There was broad consensus across stakeholder groups that screening should address all classes of substance use, rather than being confined to tobacco or alcohol alone. There were no participants who voiced a different opinion on this question. Some MDs additionally noted that screening should capture the full spectrum of severity, to identify patients with unhealthy use as well as more severe substance use disorders.

#### Should screening be universal or targeted?

##### Universal screening

Most participants were in favor of universal screening, and stated that targeted screening is likely to miss patients who have unhealthy substance use, and would be less acceptable to patients. Some patients and MAs voiced strong opinions that targeted screening could be discriminatory. One patient equated it with a ‘stop and frisk’ approach to policing, which has been criticized for targeting minority populations in NYC. These views that targeted screening may be less accurate, and that it could be less comfortable for patients who feel singled out for screening, were summarized by one patient in a focus group:

Let’s suppose they ask you if you’re on drugs, if you’re doing this, what are you doing, but they don’t ask the other one then what would…I will be thinking why he would only ask me and not him? You understand what I’m trying to say? I mean, you can’t just, you know, sit there and say, okay, you look like a drinker, you look like a smoker, you know whatever.

NY-1 Patient focus group

##### Targeted screening

While universal screening was favored by most providers, some noted that screening all patients is inefficient, and could crowd out higher value care. They noted that universal screening may not be feasible given the time limitations in primary care. In one of the MA focus groups, it was mentioned that screening older patients (over the age of 70 years) may not be necessary, since they are less likely to have substance use problems. As one provider stated,

Inherently I would think, yeah, generalized screening. But then if we are thinking within the context of primary care visit, now am I doing that at the loss of something else, other problems? And, yes, everything is important. And if I do preventative services it will be five hours a visit. So here’s where I pick and choose. I wanted the universal screening. But what gives more bang for my buck?

NY-1 Faculty MD focus group

#### How frequently should screening occur?

##### Annual screening

PCPs and MAs commented on the frequency of screening. The majority felt that screening once per year is most appropriate, and stated that annual screening is typical for other conditions. Some recommended that screening be done as part of an annual visit, though they also acknowledged that not all patients will have a dedicated annual visit for preventive care.

I think it would be great if it was a routine part of general health screening. So every time you get your annual exam, you know, we ask about exercise. We ask about nutrition. We ask about psychosocial factors. And we include alcohol, substance abuse, depression screening in all of that.

OR Faculty MD interview

##### More than once a year

A minority of participants felt that screening should occur more frequently, and possibly at every visit, because substance use behaviors can change over time. They expressed concern that substance use can escalate quickly, and that limiting screening to once per year could result in missed opportunities for early intervention with respect to new or resurgent substance use. This view was expressed by a MA, who stated:

I do think it should be more than once a year. Because you could go do through something midyear and then go see the doctor and that question doesn’t pop up. And then you’re depressed, and then it’s not being taken care of and something serious later could happen.

NY-2 Medical Assistant

#### How should screening be administered?

##### Patient self-administered screening

There was some discrepancy of opinion regarding how screening should be administered, but the majority of participants preferred a self-administered approach. Self-administered screening was favored by providers and staff because it could save time. There was also a perception that patients would be more comfortable disclosing substance use if they did not have to report it face-to-face. This view is illustrated in the following comment from a Registered Nurse:

If I’m the patient, I think I’ll be more truthful just checking it on the form rather than a medical assistant asking me. It’s kind of like if I’m taking drugs then I’ll be embarrassed telling her.

NY-1 RN focus group

##### Face-to-face screening

A minority of participants preferred that screening be done in person, while also noting that the patient’s trust in the person asking the questions is important. Some participants, including patients and providers, expressed concern over recording substance use related information in writing, and felt it would be more comfortable to talk about it than putting it down on a form. As expressed by one patient:

Because sometimes you don’t know where that form is going. You want it to be real personal. I want to talk to my doctor… I think that when people put it down on paper it becomes real.

NY-1 Patient focus group

There was also a broader discussion in some groups that medical providers spend too much time looking at the EHR, and that this makes care less personal and can be a barrier to developing patient-provider relationships. As expressed by a MA, this was a reason for preferring face-to-face screening:

Sometimes even these patients complain that the doctors are so busy with the computer, you know writing things while they’re being seen…you know seeing the patient. So I think it’s more personal when you have that contact face to face to discuss what your issues are.

NY-2 MA focus group

#### Who should administer screening, if it is done face-to-face?

There were differences of opinion regarding who in the clinic should administer screening. Among MDs, most preferred that screening be done by ancillary staff in the clinic, and usually identified screening as a MA role. In the participating clinical sites, it was common practice for the MAs to deliver depression screening, and substance use screening was perceived as being similar. For the minority of PCPs who believed that screening should be administered by physicians, reasons included that the MAs are already quite burdened, variation in the quality of screening delivered by MAs, and beliefs that patients have greater trust in their physicians than in other members of the care team. They also noted that because it is ultimately the provider who needs to address a positive screen, they may want to ask the questions themselves. Concerns about the quality of MA-delivered screening were expressed by one NY physician:

That’s how…is the MA doing it? Is the MA looking at the screen and just asking the questions and not making eye contact and just checking off the boxes? ..Unless, again, it’s like you know which MA has done it. And you know what they’re like and what that rapport is…

NY-2 Faculty MD focus group

In contradistinction to the opinion of most PCPs, the majority of MAs and patients stated a preference for PCPs to deliver screening, if it is to be administered face-to-face. They felt that PCP screening was more likely to be accurate, and would give patients an opportunity to discuss or clarify their responses with the provider. Some patients felt that their physician was better able to interpret their body language and engage them in conversation about their responses. MAs felt that patients would be more comfortable giving sensitive information only to their PCP. Some patients also worried that information could be lost or distorted if it was collected by the MA and then passed along to the PCP. This viewpoint is captured in a statement from one patient:

And then sometimes it can also be a communication breakdown between the medical assistant and getting it to the doctor. I would be worried that my doctor wouldn’t get it the way I would deliver it.

NY-1 Patient focus group

#### When should screening occur?

##### Prior to the medical encounter

Most participants felt that screening should be done prior to the medical encounter, either in the waiting room or before the patient arrives in clinic. A number of providers noted that the most preferred option would be for screening to be completed electronically, ideally through the patient portal while the patient is at home, and then integrated into the EHR prior to the visit.

What I would want to do is all the questionnaires would be done online. As much as can be done before the appointment would be put on the tablet. So they would update their medication list online. They would do all their questionnaires. So they would upgrade all their information. And it would all be done and ready to go for the MA and the provider. So that would be my ideal world.

OR Faculty MD interview

##### During the clinic visit, in the exam room

Patients and MAs discussed the timing and location of screening during course of the medical visit, and did not bring up the possibility of pre-visit screening. Some expressed concerns about privacy, and felt screening was best performed in the exam room, and not in the waiting area. Patients and MAs were less likely than MDs to discuss electronic approaches to screening. Some expressed pessimism about the feasibility or acceptability of electronic screening for patients, particularly those who are older or less familiar with computers. As one patient stated:

I’d rather do it on paper. Or if I’m not capable, have someone verbally ask me. I don’t like computers. I’m not going to get into that. I wouldn’t answer the questions if it was through the computer. That’s me. And I find that a lot of my friends, the seniors, prefer the paperwork or someone to ask them, like a receptionist or something, you know.

NY-1 Patient focus group

## Discussion

Through interviews with key clinical stakeholders, this study characterized current substance use screening practices, barriers to screening, and recommendations for its implementation in primary care clinics. Our approach is unique in capturing the views of patients, as well as those of primary care providers, residents, MAs, and RNs. To be successful in practice, screening must be acceptable to all of these groups. By including participants from two health systems that differ markedly both in their geography, their health care environment, and their experience with SBIRT, we captured diverse views.

In the KTA framework, essential early steps in implementation are to identify, address barriers, and adapt knowledge to the local context. This process is important for guiding implementation strategies. Over the past decade, many attempts to introduce screening and brief intervention (SBI) have faced challenges in their implementation, or have not been sustained in the absence of grant funding [24, 38–41]. We considered these interviews, which were conducted as the initial phase of a larger implementation study, to be essential for designing a screening strategy that would address as many stakeholder concerns as possible, and thus be more likely to be adopted and sustained.

Participants from all stakeholder groups felt strongly that screening for substance use is important for the quality and safety of medical care, and is an essential part of the patient’s medical history. In order to make accurate diagnoses, manage other medical conditions, and provide appropriate preventive care, it is important for medical providers to know about a patient’s substance use [42]. This is a benefit of screening that is frequently overlooked in current discussions about the efficacy of SBIRT programs for reducing drug use. Current USPSTF guidelines recommend alcohol SBI for adults in primary care settings (Grade B recommendation) [13], but the evidence is considered insufficient to support a similar recommendation for drugs [43]. Recent clinical trials of SBI have had mixed results, with two studies demonstrating no impact on drug consumption [44, 45], and one study showing short-term reductions [46]. Notably, the study by Gelberg et al. that did show reductions in drug use [46] employed patient self-administered screening and provided PCPs with a prompt and decision support encouraging them to offer very brief advice. This approach could address some of the important barriers identified by participants in our study, including patient fears about how providers will react, and provider lack of time and knowledge to intervene on unhealthy substance use. While more research is needed on primary care-based approaches to identifying and effectively addressing drug use [47], this does not diminish the importance of screening’s role in informing clinical care for other health problems.

Importantly, we captured the views of patients on primary care-integrated screening and interventions. While there is a rich literature on medical provider attitudes in this area [48–56], much less is known about patient attitudes [57, 58]. One study in a Colorado health system, conducted by Rahm et al., interviewed primary care stakeholders, including patients as well as PCPs and clinical staff, about SBIRT implementation [58]. Similar to the views of patients captured in our interviews, patients in the Colorado study endorsed universal screening as part of routine care, felt that it could help open up patient-provider discussions about substance use, and expressed concerns about the confidentiality of this information when documented in the EHR. Our patient interviews also identified a number of additional individual-level barriers to screening, including fear of the medical provider’s reaction, and the stigma associated with SUDs, which patients felt were not treated like other medical conditions. These concerns were similarly expressed by primary care patients in one prior study, also conducted in New York City [57], which found that patients considered substance use to be highly stigmatized, and felt that patients would only share this information with trusted primary care providers.

Providers have stated concerns about patients being uncomfortable with screening in previous studies evaluating screening implementation efforts [41, 59], but patient voices on this subject have not previously been well articulated. While some patients may feel comfortable raising the subject of substance use [51], our interviews indicate that many will not disclose this information unless they are reassured that they will not feel ‘judged’ by their providers or experience negative consequences from doing so. Our findings are consistent with previous literature showing that substance use disorders are among the most severely stigmatized health conditions [60]. This is particularly concerning in light of evidence that patients who feel stigmatized by providers have poor treatment outcomes [61, 62].

An additional theme from our patient interviews, which has not been highlighted in the screening implementation literature, is that patients lack confidence in their medical providers’ ability to effectively address substance use. In the face of stigma and expectations of negative repercussions from disclosing substance use, patients need to feel that there is benefit to doing so. This benefit can only be realized if medical providers are prepared to deliver appropriate medical care and treatment interventions to patients with unhealthy substance use. While more research on patient attitudes toward screening is needed, our findings point to the importance of articulating the value and purpose of screening to patients, informing patients about exactly how their substance use information will be used, and addressing perceived negative attitudes and lack of knowledge among medical providers and staff as being highly important to the success of any screening program.

Our interviews with providers reiterated many of the individual- and systems-level barriers that have been found in prior research [48–56, 63]. Medical providers, including PCPs and MAs, repeatedly raised lack of time and competing demands during the primary care visit, and expressed concerns that physicians lack knowledge about substance use and addiction. Perhaps related to lack of knowledge about the spectrum of substance use that is typically identified via primary care screening, providers in our study seemed to conflate a positive screen with a need for addiction treatment, which would be accomplished by making a referral for specialty care. Providers voiced frustration about lack of access to treatment resources, and not knowing how to refer patients to treatment programs, but there was almost no mention of the potential role of PCPs themselves in providing treatment for substance use disorders. This finding echoes another recent primary care study, which found that PCPs frequently believed that treatment for alcohol use disorder requires intensive counseling resources and that specialty care is most effective [56]. More focused efforts are needed to engage patients in treatment, including primary care-based SUD treatment (e.g., office-based opioid and alcohol pharmacotherapy) or the use of on-site behavioral health providers who can assess and engage patients, and make warm hand-offs to specialty care. Our study indicates that having effective approaches to delivering treatment is important for cultivating PCP support for a substance use screening program.

## Limitations

Our study has some limitations. While we included sites from very different parts of the U.S., both were academic medical centers located in urban areas. Individuals from rural areas, or in smaller community primary care practices, may have different views on substance use screening. Most interviews were done at the New York site, and we were not able to conduct any patient interviews or focus groups in Oregon, which gave us a more limited picture of stakeholder attitudes from this health system. PCPs in our sample included both residents and faculty, but all were physicians. This reflects the characteristics of our sites, in which the majority of primary care providers, and all individuals identified as having SBIRT or HIT expertise, were MDs. Licensed providers with different training, such as nurse practitioners or Doctors of Osteopathy, may have different views about screening. While we consider the inclusion of patients to be an important strength of the study, it proved difficult to recruit younger patients, and so their views may be underrepresented. This could be important, because younger patients may have higher rates of substance use, and greater concerns about issues such as the impact of screening on their employment or health insurance. Interviews were conducted only in English, which also excluded some patients. Because our objective was to capture the views of a general primary care patient population, we have little representation of patients with current alcohol and drug use disorders. Our interviews were conducted by the study PI (in New York) or the site Lead Investigator (in Oregon), which has the potential to introduce social desirability bias. However, we did not see direct evidence of this in the interviews, in which many participants spoke critically about the current system of care and about substance use screening and interventions.

Finally, we found that stigma and negative attitudes toward substance use were common themes in our interviews, particularly in the patient focus groups. While the KTA framework includes individual attitudes as ‘barriers to knowledge use,’ being a process model it focuses less on the factors that underlie negative attitudes, or on how attitudes may change through the process of implementing a new practice. Employing an additional theoretical model that more explicitly explores individual attitudes and behavior change may have been useful for fully exploring these themes.

## Conclusion

This qualitative study can inform the design of substance use screening programs in primary care practices. Based on our findings, we have designed and are now testing a strategy that seeks to optimally utilize existing staff and resources to deliver screening in the participating clinics. We are implementing validated brief screening questionnaires, administered annually to all patients, using a patient self-administered approach when possible. Screening results will be paired with EHR-integrated clinical decision support to assist primary care providers in conducting a brief counseling intervention. We have improved the system for linking patients with high-risk substance use to care by identifying a clear process for referring patients to the clinic’s existing social workers, training the social workers in brief intervention, and improving their knowledge of referral sources for addiction treatment. We are hopeful that educating medical providers about substance use and interventions will begin to address providers’ negative attitudes toward patients with unhealthy alcohol and drug use, but more work is likely needed to address stigmatizing beliefs among providers and clinical staff. To directly address patient concerns about stigma, we plan to use signage and consistent language to communicate to patients that screening is universal, and is part of routine medical care.

Our findings provide general guidance regarding key elements of a screening implementation approach for primary care settings. Screening programs should clearly communicate the goals of screening to patients and seek to proactively counteract stigma, address staff concerns regarding time and workflow, and provide education as well as treatment resources to primary care providers. Without a significant infusion of resources (to support, for example, longer primary care visits, or more behavioral health staff), no screening strategy will be able to address all of the barriers that were identified. Patient concerns about the confidentiality of their screening results, and about how their providers will react, remain as important challenges to screening in medical settings. However, our understanding of stakeholder views can inform substance use screening implementation efforts by identifying important barriers that need to be acknowledged, and addressed to the extent possible, when initiating a screening program.

## Additional file

**Additional file 1.** Interview Guides.

## Abbreviations

CCO: Coordinated Care Organization
CTN: Clinical Trials Network
EHR: electronic health record
KTA: Knowledge to Action (implementation framework)
MA: medical assistant
MD: medical doctor
NIDA: National Institute on Drug Abuse
PCP: primary care provider
PI: principal investigator
RN: registered nurse
SBI: screening and brief intervention
SBIRT: screening, brief intervention, and referral to treatment
USPSTF: United States Preventive Services Task Force
