# Supplementary material for: Barriers and facilitators affecting the implementation of substance use screening in primary care clinics: a qualitative study of patients, providers, and staff
Source: Addict Sci Clin Pract. 2018 Apr 9;13:8. doi: 10.1186/s13722-018-0110-8 (PMC5890352; doi:10.1186/s13722-018-0110-8)
Supplement: Supplementary file 1 — Additional file 1. Interview Guides. [file 13722_2018_110_MOESM1_ESM.doc]

**MODERATOR GUIDE – Patient Focus Groups**

Preamble/Opening Statements

- Thank you very much for coming to the focus group today. We would like to learn more about how to ask patients about their drug and alcohol use.
- This group is an opportunity for us to learn from you. I’m hoping that you will teach me about your own experiences and opinions as patients – you are the experts here.
- You were selected for this group because all of you are patients in this primary care clinic.
- The focus here is not your own substance use, but rather how you think that patients in general would feel about being asked about their substance use.

Substance use screening and barriers : What we’re talking about today is the idea of screening for substance use, including tobacco, alcohol, and drugs. When I say ‘screening,’ I’m generally talking about asking patients questions about their substance use. Some people think that this something that should be part of regular primary care. Others say that it doesn’t belong in the medical clinic. I’m interested in hearing what you think.

1. Do you think it’s important to ask patients about their alcohol or drug use?
2. Should all patients be asked, or should it be more targeted?
3. Why would a clinic want to ask patients about their substance use? Who would use this information?
4. How do you think patients would feel about completing substance use screening questionnaires in your health center?

- Would it make them less likely to come to the clinic?
- What if they were a heavy drinker or a cocaine user… would that change how they would feel about answering questions about their substance use?

1. If patients are asked about their drug and alcohol use by their doctor, do you think they feel singled out – like they’re being asked because their doctor thinks they have a substance abuse problem?
2. Would patients answer honestly? What would make them be honest or not?
3. Do you think patients would prefer answering questions about substance use on their own (on paper/computer), or face to face with an interviewer?
4. How do you think patients feel about information about their substance use being part of their medical record?

- Do you think this information is already part of the medical record?

1. If a patient has a drug or alcohol problem, what do you think they want from their medical provider?

- Do you think they would want to discuss it?
- What would they expect their provider to do?

1. Do you think medical providers know how to help patients who have unhealthy drug or alcohol use?

- Are providers comfortable discussing it?
- Are there any ways the provider can help?

Closing items

1. Let’s say we were planning to start screening all patients for substance use in this health center. What do you think is the single most important thing we need to consider before doing that?

**MODERATOR GUIDE – Medical Staff Focus Groups**

Preamble/Opening Statements

- Thank you very much agreeing to participate in this focus group.
- The purpose of this discussion is to better understand the potential barriers and facilitators to introducing substance use screening in primary care practices in general, and how it might best be done in this clinic more specifically.
- You were selected for this focus group because you are a member of the medical staff in this health center, which has agreed to participate in a study about implementing substance use screening in primary care settings.

Substance use screening and barriers : What we’re talking about today is the idea of screening for substance use, including tobacco, alcohol, and drugs. When I say ‘screening,’ I’m generally talking about asking patients questions about their substance use. Some people think that this something that should be part of regular primary care. Others say that primary care clinics are already too busy, and they question whether providers should be asked to screen for substance use, on top of everything else they’re already doing. I’m interested in hearing your thoughts on this.

1. Do you think it’s important to identify unhealthy alcohol or drug use in primary care clinics?

- Is it something that should needs to be identified through screening, or do you already know which patients have a problem? Or can you wait for patients to bring it up?

1. Should all patients be screened, or should it be more targeted?

- If it was targeted, what are the characteristics of patients who should be screened?

Adapting to the local context: Now I would like to shift the focus to this clinic, and talk with you about how screening could best be implemented here.

1. Are there processes already in place here to screen for substance use – including tobacco?
2. If screening was to be implemented in this clinic, whose responsibility should it be?
3. Who do you think should administer screening (patient, medical assistant, nurse, MD, etc.)?
4. Where should screening occur? Should it be done before the patient sees their doctor, or as part of the medical visit? What about asking patients to do it from home, or in the waiting room?
5. What substances should be included (tobacco, alcohol, illicit drugs, non-medical use of prescription drugs)?
6. Should it be collected on paper or electronic forms?
7. How long should screening take?
8. What would be some good strategies for fitting screening into your regular workflow?

Comfort with screening

1. How comfortable do you think medical staff are, in general, with asking patients about their drug and alcohol use?

- Is this different from other types of questions you already ask?

1. Do you think staff would more comfortable asking the patients directly, or having patients fill out a screening form on their own?
2. How do you think patients would feel about completing substance use screening questionnaires in your health center? Would they answer honestly? Would they be more comfortable with a self-administered questionnaire, or a face-to-face approach?

Closing items

14) Let’s say we were planning to start universal screening for substance use in this health center. What do you think is the single most important thing we need to consider before doing that?

**MODERATOR GUIDE – Medical Provider Focus Groups**

Preamble/Opening Statements

- Thank you very much agreeing to participate in this focus group.
- The purpose of this discussion is to better understand the potential barriers and facilitators to introducing substance use screening in primary care practices in general, and how it might best be done in this clinic more specifically.
- You were selected for this focus group because you are a medical provider in this health center, which has agreed to participate in a study about implementing substance use screening in primary care settings.

Substance use screening and barriers : What we’re talking about today is the idea of screening for substance use, including tobacco, alcohol, and drugs. When I say ‘screening,’ I’m generally talking about asking patients questions about their substance use (not, for example, urine toxicology tests). Some believe that substance use screening should be part of regular primary care. Others say that primary care clinics are already too busy, and they question whether providers should be asked to screen for substance use, on top of everything else they’re already doing. I’m interested in hearing your thoughts on this.

1. Do you think it’s important to identify unhealthy alcohol or drug use in primary care?

- Is it something that should needs to be identified through screening, or can you wait for patients to bring it up?

1. Should all patients be screened, or should it be more targeted?

- If it was targeted, what are the characteristics of patients who should be screened?

1. How do you think patients would feel about completing substance use screening questionnaires in your health center? Would they answer honestly? Would they be more comfortable with a self-administered questionnaire, or a face-to-face approach?

Adapting to the local context: Now I would like to shift the focus to this clinic, and talk with you about how screening could best be implemented here.

1. Are there processes already in place here to screen for substance use – including tobacco?
2. If screening was to be implemented in this clinic, whose responsibility should it be?
3. Who should administer screening (patient self-administered, medical assistant, MD, etc.)?
4. What substances should be included (tobacco, alcohol, illicit drugs, non-medical use of prescription drugs)?
5. How long should screening take?
6. Should substance use screening stand alone or be incorporated into a more comprehensive behavioral health screen, or a preventive health screen? What should be included with it? What are the pros and cons of a stand-alone versus combined approach to screening?
7. Should it be collected on paper or electronic forms?
8. What would be some good strategies for fitting screening into your regular workflow?
9. Where should screening occur? Should it be done before the patient sees you, or as part of the medical visit? What about asking patients to do it from home, or in the waiting room?
10. If screening was done before the patient sees you, what would be the best way to get this information to providers? Where should it be located in the EHR, can clinical alerts be used?

Clinical Decision Support

1. What type of information do medical providers need in order to effectively and efficiently intervene on unhealthy substance use?
2. How do you feel about Clinical Decision Support tools? What would make them more or less useful here?

Other Support

1. What type of support would you need in order to more effectively address substance use in your patients? What has been helpful in helping you to address other medical conditions, and do you think it could apply to substance use?

Closing items

1. Let’s say we were planning to start universal screening for substance use in this health center. What do you think is the single most important thing we need to consider before doing that?

**INTERVIEW GUIDE – Medical Provider Interviews**

Preamble/Opening Statements

- Thank you very much agreeing to participate in this interview.
- You were selected for this interview because you are a medical provider in a health center that has agreed to participate in a study about implementing substance use screening in primary care settings.

Substance use screening and barriers : It feels like primary care providers are always being asked to do more, and in less time – especially with respect to prevention practices. In this context, many people question whether providers should be asked to screen for substance use, on top of everything else they’re already doing.

1. Do you think it’s important to identify unhealthy alcohol or drug use in primary care?

- If a patient doesn’t ask you about substance use, how often do you bring it up?
- How much of a priority is screening for alcohol and drug use for you, with your patients?
- What circumstances make it a higher/lower priority?

1. How often do you think primary care providers identify drug or alcohol use in their patients?

- What is your own practice for identifying substance use in your patients?
- Do you think patients should be systematically screened for substance use? Should screening be targeted or universal?

1. What do you see as the barriers to screening patients for substance use in primary care?

- Do you have any thoughts on how these barriers might be overcome?

Readiness to address substance use

1. How *comfortable* are you discussing substance use with patients? What would make you feel more comfortable?
2. How *prepared* do you feel to discuss substance use with patients? Are you equally prepared to discuss alcohol and drugs? Is there anything that would make you feel better prepared?
3. How *effective* do you feel you are at addressing substance use with patients? What type of support would you need in order to more effectively address substance use in your patients? What types of tools or support has been helpful in helping you to address other medical conditions, and do you think it could apply to substance use?
4. What if you received the results of alcohol screening for one of your patients, indicating that the patient had hazardous drinking. Would you want to bring it up with them? What type of information or support would you want to have at your fingertips?
5. Do you think that more training or education on substance use would be helpful for you? What would be the best way of providing this?

Screening approaches

1. How do you think *providers* would feel about receiving results of a screening questionnaire that patients filled out on their own? Can you tell me about any pros and cons of this approach?
2. How do you think *patients* would feel about completing substance use screening questionnaires in your health center? Do you think they would complete the questionnaires? Would they answer honestly? Would they be more comfortable with a self-administered questionnaire, or a face-to-face approach?

Closing items

1. Let’s say we were planning to start universal screening for substance use in this health center. What do you think is the single most important thing we need to consider before doing that?
